# Systematic identification of long intergenic non-coding RNAs expressed in bovine oocytes

**DOI:** 10.1186/s12958-020-00573-4

**Published:** 2020-02-21

**Authors:** Jian Wang, Prasanthi P. Koganti, Jianbo Yao

**Affiliations:** Division of Animal and Nutritional Sciences, West Virginia University, Morgantown, WV 26506 USA

**Keywords:** Cattle, Oocyte, lncRNA, lincRNA

## Abstract

**Background:**

Long non-coding RNAs (lncRNAs) are key regulators of diverse cellular processes. Although a number of studies have reported the identification of bovine lncRNAs across many tissues, very little is known about the identity and characteristics of lncRNAs in bovine oocytes.

**Methods:**

A bovine oocyte cDNA library was constructed and sequenced using the Illumina HiSeq 2000 sequencing system. The oocyte transcriptome was constructed using the ab initio assembly software Scripture and Cufflinks. The assembled transcripts were categorized to identify the novel intergenic transcripts, and the coding potential of these novel transcripts was assessed using CPAT and PhyloCSF. The resulting candidate long intergenic non-coding RNAs (lincRNAs) transcripts were further evaluated to determine if any of them contain any known protein coding domains in the Pfam database. RT-PCR was used to analyze the expression of oocyte-expressed lincRNAs in various bovine tissues.

**Results:**

A total of 85 million raw reads were generated from sequencing of the bovine oocyte library. Transcriptome reconstruction resulted in the assembly of a total of 42,396 transcripts from 37,678 genomic loci. Analysis of the assembled transcripts using the step-wide pipeline resulted in the identification of 1535 oocyte lincRNAs corresponding to 1183 putative non-coding genes. A comparison of the oocyte lincRNAs with the lncRNAs reported in other bovine tissues indicated that 970 of the 1535 oocyte lincRNAs appear to be unique to bovine oocytes. RT-PCR analysis of 5 selected lincRNAs showed either specific or predominant expression of 4 lincRNAs in the fetal ovary. Functional prediction of the oocyte-expressed lincRNAs suggested their involvement in oogenesis through regulating their neighboring protein-coding genes.

**Conclusions:**

This study provides a starting point for future research aimed at understanding the roles of lncRNAs in controlling oocyte development and early embryogenesis in cattle.

## Background

Over the past decade, genome-wide transcriptional studies have discovered that the vast majority of the mammalian genome (up to 80%) is transcribed, while only 2–3% of the mammalian genome is transcribed into protein-coding RNAs (mRNAs) [1, 2]. The transcripts that function as non-translated RNA molecules are called non-coding RNAs (ncRNAs). Recent advances in transcriptome sequencing have allowed for the discovery of a new class of ncRNAs that are generally longer than 200 nucleotides, known as long non-coding RNAs (lncRNAs). LncRNAs transcribed from intergenic region are referred to as lincRNAs. Like protein-coding genes, lncRNAs are usually 5′ capped, 3′ polyadenylated and alternative spliced [2]. The study of lncRNAs is now focusing on understanding their functions, revealing that lncRNAs play various roles in diverse biological processes, including regulation of epigenetic marks and gene expression at different levels, as well as protein post translational modification [3]. According to the genomic position of the loci from which they are transcribed and their proximity to protein coding genes in the genome, lncRNAs can be divided into five categories: sense, antisense, bidirectional, intronic and intergenic lncRNAs [3]. In the past few years, an increasing number of lncRNAs have been reported in eukaryotic organisms, ranging from nematodes to human [4–10]. So far, three major lncRNA database including LNCipedia [11], GENCODE and NONCODE [12] have archived more than 100,000 human lncRNA genes.

Cattle (*Bos taurus*) is one of the most commonly raised livestock for meat, milk and other dairy products. As lncRNAs play a key role in the regulation of gene expression, it is important to identify and characterize bovine lncRNAs. To date, a number of studies have reported the identification of bovine lncRNAs across many tissues [13–16]. For example, a total of 449 putative lncRNAs were identified using publicly available bovine expressed sequence tag sequences [13]. Over 4000 lncRNAs were predicted from bovine skin RNA-Seq data [15] and a stringent set of 584 lincRNAs was identified in bovine muscle [16]. More recently, Koufariotis et al. (2015) reported a total of 9778 lncRNAs identified through analysis of RNA-Seq data across 18 bovine tissues [17]. However, very little is known about the identity and characteristics of lncRNAs in bovine oocytes. The developmental competence of an oocyte, also known as egg quality, is defined as the ability of the egg to be fertilized and subsequently develop into a normal embryo. Mammalian oocytes become transcriptionally silent following germinal-vesicle breakdown, so the final stages of oocyte maturation and early embryo development depend on stored transcripts. Several studies have reported that lncRNAs play critical roles in the embryonic stem cell regulatory network [6, 18–20]. For instance, more than 100 lincRNA promoters were identified to be bound by stem cell factors such as OCT4 and Nanog [20]. Therefore, the study of lncRNAs in bovine oocytes could help us understand the early events of embryonic development. In this study, we described a comprehensive catalogue of putative lincRNAs expressed in bovine oocytes. We also compared our results to those from other bovine studies, assessed the tissue specificity of each lincRNA and performed functional prediction for oocyte-specific lincRNAs.

## Methods

### RNA-sequencing of bovine oocytes

Bovine germinal vesicle (GV) and metaphase II (MII) stage oocytes were obtained using procedures as described previously [21]. Total RNA was isolated from a pool of 20 GV and 20 MII stage oocytes using the RNAqueous™-Micro kit (Thermo Fisher Scientific, Waltham, MA). The RNA was subsequently converted to cDNA with linear amplification using the Ovation RNA-Seq System (NuGEN Technologies, Inc., San Carlos, CA) according to the manufacturer’s instructions. The cDNA sample was sent to the W.M. Keck Center for Comparative and Functional Genomics at the University of Illinois at Urbana-Champaign (Urbana, IL) where RNA-Seq library was constructed and sequencing was performed using the Illumina HiSeq 2000 sequencing system. A total of 85 million reads were yielded, and the reads were paired and both lengths were 100 bp.

### Publicly available annotations

Protein-coding genes were downloaded from UCSC genome browser [22] and Ensembl genome browser [23]. All known noncoding genes were downloaded from Ensembl genome browser [23] and NONCODE database [12].

### RNA-Seq reads mapping and assembly

After trimming adaptor sequences and filtering rRNAs and ambiguous and low-quality bases, a total of 78 million pair-end reads were obtained. Spliced read aligner TopHat2 was used to align all clean reads to the bovine genome (UMD3.1) using the default parameters. Aligned reads from TopHat2 were assembled into transcriptome by Scripture [6] and Cufflinks [24]. Both assemblers use spliced read information to determine exon connectivity; however, with two different approaches. Cuffcompare [24] was used to determine a unique set of isoforms assembled from both assemblers for further lincRNA identification.

### LincRNA identification pipeline

Identification of each transcript as either coding or noncoding was performed using a step-wise pipeline to filter out the transcripts that had a high chance of being protein coding. First, all transcripts that had exon overlapping a transcript from any of the following sets were eliminated: coding genes annotated in UCSC, RefSeq and Ensembl, and microRNAs, tRNAs, snoRNAs and rRNAs annotated in Ensembl. Second, coding potential of each candidate transcript was assessed using PhyloCSF [25] and CPAT [26]. PhyloCSF uses a multispecies nucleotide sequence alignment to estimate the degree of evolutionary pressure on sequence substitutions to preserve an open reading frame. PhyloCSF was run using multiple sequence alignment of 5 mammalian genomes including cow, human (hg19), mouse (mm10), rat (m5) and dog (canfam3). CPAT assesses the coding potential of a transcript based on the length and quality of open reading frame (ORF) with premise that true protein coding gene is more likely to have a long and high-quality ORF. Third, to evaluate which of the remaining transcripts contains a known protein coding domain, HMMER-3 [27] was used to identify transcripts translated in all three possible frames having homologs with any of the 31,912 known protein family domains in the Pfam database (release 24; both PfamA and PfamB). All transcripts with a Pfam hit were excluded. Finally, putative protein-coding RNAs were filtered out by applying a maximal ORF length threshold. All transcripts with a maximal ORF > 100 amino acids were excluded.

### Tissue specificity score

To evaluate tissue specificity of a transcript, an entropy-based metric that relies on Jensen-Shannon (JS) divergence was used to calculate specificity scores (0 to 1). A perfect tissue-specific pattern is scored as JS = 1, which means a transcript is expressed only in one tissue [28].

### RT-PCR analysis of lincRNA expression

Bovine tissue samples including fetal ovary, adult ovary, fetal testis, adult testis, liver, kidney, muscle, heart, thymus, spleen, adrenal, cortex, pituitary, stomach, intestine and lung were collected at a local slaughterhouse. Granulosa and theca cells were isolated from antral follicles according to a previously established method [29]. Total RNA was isolated from these tissues using TRIzol reagent (Invitrogen, Carlsbad, CA) and treated with DNase (Promega, Madison, WI) according to the manufacturers’ protocols. Total RNA was converted cDNA using oligo (dT)_18_ primer and Superscript III reverse transcriptase (Invitrogen, Carlsbad, CA). The cDNA was used for PCR amplification using lincRNA-specific primers (Table 1). The PCR was performed using 35 cycles of 94 °C for 40 s, 59 °C for 40 s and 72 °C for 40 s, and a final extension at 72 °C for 10 min. Bovine ribosomal protein L19 (RPL19) was used as a control for RNA quality.

**Table 1:** Primers used in this study

Primer name	Primer sequence (5′-3′)	Application
lincRNA.41165-F	TGAAGAACCATTGCTCGAGAG	RT-PCR
lincRNA.41165-R	GTATGGTAACTTATTATCTCAGG	RT-PCR
lincRNA.25823-F	TTCAATGCCGGTTCTTCATGC	RT-PCR
lincRNA.25823-R	CAAGTCCCTGCCAAGACATTG	RT-PCR
lincRNA.2160-F	CCAACAGCTCATCTGTCAATT	RT-PCR
lincRNA.2160-R	GAACTGTTTCCTGCTGTTTGC	RT-PCR
lincRNA.17345-F	CAGCTTTGAAGTCACTTCAGG	RT-PCR
lincRNA.17345-R	AAACATCTTCACTGAGTCTGG	RT-PCR
lincRNA.25909-F	AGATTGCTGCAAACTCTGCAG	RT-PCR
lincRNA.25909-R	ATCCAGTAGGCATTCATTGAG	RT-PCR
RPL19-F	GAAATCGCCAATGCCAACTC	RT-PCR
RPL19-R	GAGCCTTGTCTGCCTTCA	RT-PCR

## Results

### Transcriptome reconstruction of bovine oocytes

A total of 85 million raw reads were generated from sequencing of the bovine oocyte library. These reads were paired and both lengths were 100 bp. After quality control, 78 million clean reads were obtained. All clean reads were further mapped to bovine genome (UMD3.1) using TopHat2 [30]. 78.4% (61 million) of the clean reads were aligned onto the bovine genome, and 82% of the mapped reads were aligned concordantly. The mapping ratio was similar to those obtained in other RNA-Seq studies in cattle [31–36]. We then used ab initio assembly software Scripture [6] and Cufflinks [24] to reconstruct the transcriptome based on the read-mapping results. Transcripts reconstructed by these two assemblers were merged into a combined set of transcripts using the Cuffcompare utility provided by Cufflinks, resulting in the assembly of a total number of 42,396 transcripts from 37,678 genomic loci.

All assembled transcripts were categorized using the bovine genome annotation obtained from UCSC and Ensembl genome browser (Table 2). Approximately 40% of the transcripts correspond to already annotated transcripts. Notably, ~ 17% (7106) of the transcripts correspond to novel isoforms of known genes (“j” class), indicating that a large number of new transcript isoforms have yet to be annotated or the bovine genome remains poorly annotated. Interestingly, more than 19% (8336) of the transcripts were categorized as unknown intergenic transcripts (“u” class). After removing all single exon unknown intergenic transcripts, 2552 multi exon transcripts that have class code of “u” were selected for lincRNA identification.

**Table 2:** Statistical summary of bovine oocyte sequencing, assembly and annotation

RNA-sequencing	
Number of raw reads	84,860,000
Number of clean reads	78,250,146 (94%)
Number of mapped reads	61,494,822 (78.4%)
Number of concordant pair alignment	50,425,754 (82%)
Number of transcripts in each transfrag class	
=	2165
1c	7408
j	7106
e	582
i	4792
o	1699
p	2574
u	8336
x	462
s	4
LincRNA identification	
Number of novel transcripts with multiple exon	2552
Number of transcripts without coding potential	1627
Number of lincRNA (protein domain filter)	1535 (1183 loci)
Average length	782 bp
Number of average exon	2.6

=: Complete match of intron chain. c: Contained by a reference transcript. j: Potentially novel isoform. e: Single exon transfrag overlapping a reference exon and at least 10 bp of reference intron. i: Intronic transcript. o: Generic exonic overlap with a reference transcript. p: Possible polymerase run-on fragment. u: Unknown, intergenic transcript. x: Exonic overlap with reference on the opposite strand. s: Transcript overlap with reference intron on the opposite strand

### Identification of putative lincRNAs

To identify lincRNAs, we first analyzed the coding potential of all 2552 novel intergenic transcripts using CPAT [26] and PhyloCSF [25]. PhyloCSF scores were first calculated for the 2552 putative multi exon intergenic transcripts. All transcripts with a negative score were retained as potential non-coding candidates. In addition, CPAT was also used to assess the coding potential for all 2552 transcripts. To determine the optimum cut-off value, CPAT was trained using a set of 10,000 bovine CDS from Refseq, a set of 3650 ncRNAs from Ensembl and a set of 6350 intron sequences from Refseq. A cut-off value of 0.348 was selected. Transcripts predicted by both CPAT and PhyloCSF as non-coding RNAs were selected as potential bovine lincRNAs. This procedure identified 1627 transcripts from 1249 different genome loci. Finally, we scanned each of these transcripts and evaluated whether it contained any of the known protein coding domains in the Pfam database. This step filtered out 92 transcripts and resulted in 1535 bovine lincRNAs corresponding to 1183 putative non-coding genes (Additional file 1: Table S1).

**Fig. 1:**
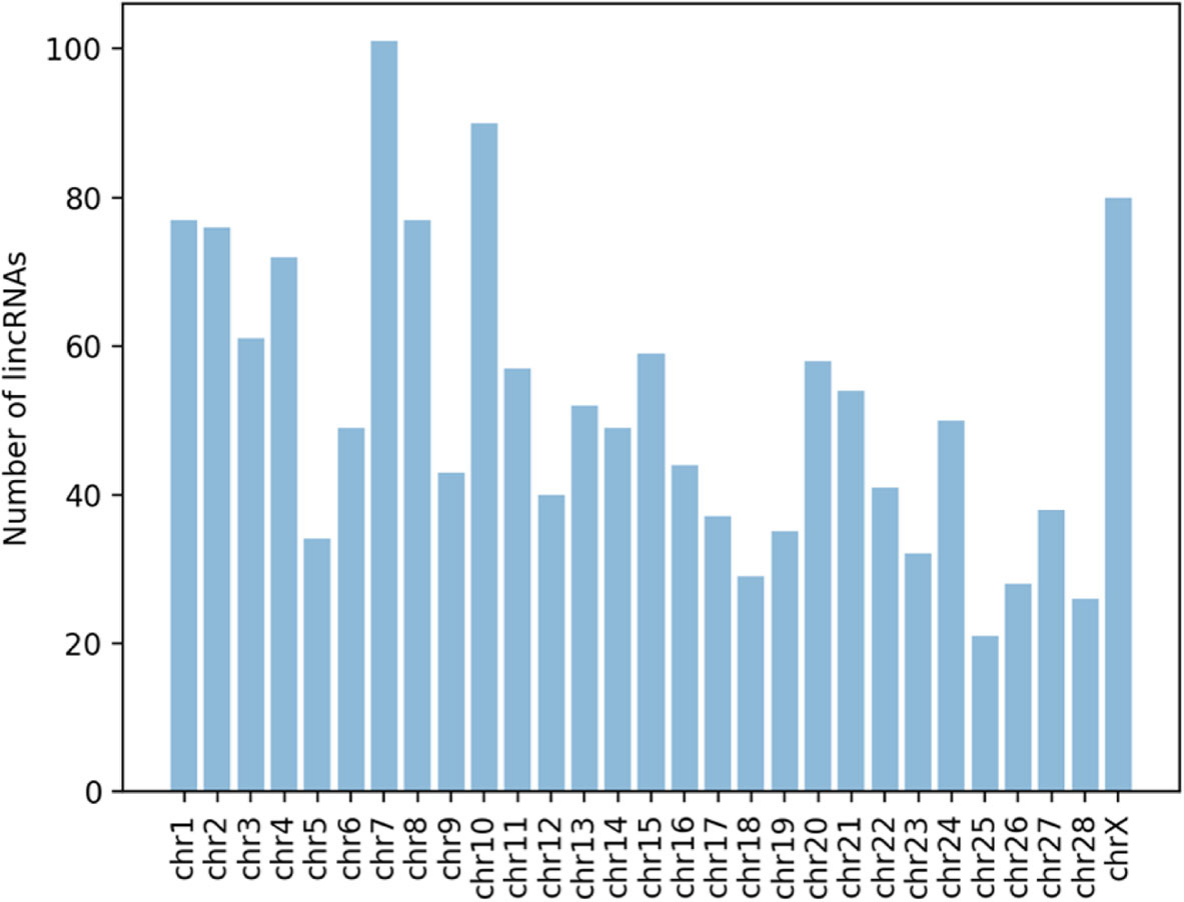
Distribution of bovine oocyte lincRNAs on different chromosomes. The X-axis represents different chromosomes and the Y-axis represents the number of lincRNAs

Previous studies in mammals have shown that lncRNAs are shorter, and have fewer exon number compared with protein-coding genes [6]. In the present study, the mean length and average exon number of bovine oocyte lincRNAs are 782 ± 580 nt and 2.6 ± 0.8 exons, respectively, which are similar to those of human (~ 1000 nt and 2.9 exons) [28] and zebrafish (~ 1000 nt and 2.8 exons) [37] lncRNAs. The genome distribution of the bovine oocyte-expressed lincRNAs was also investigated. As shown in Fig. 1, chromosome 7 has the greatest number of bovine oocyte lincRNAs, followed by chromosome 10, 1, X, 8 and 2, whereas chromosome 15 has the least number of lincRNAs.

### Comparative analysis with bovine lncRNAs from similar studies

**Fig. 2:**
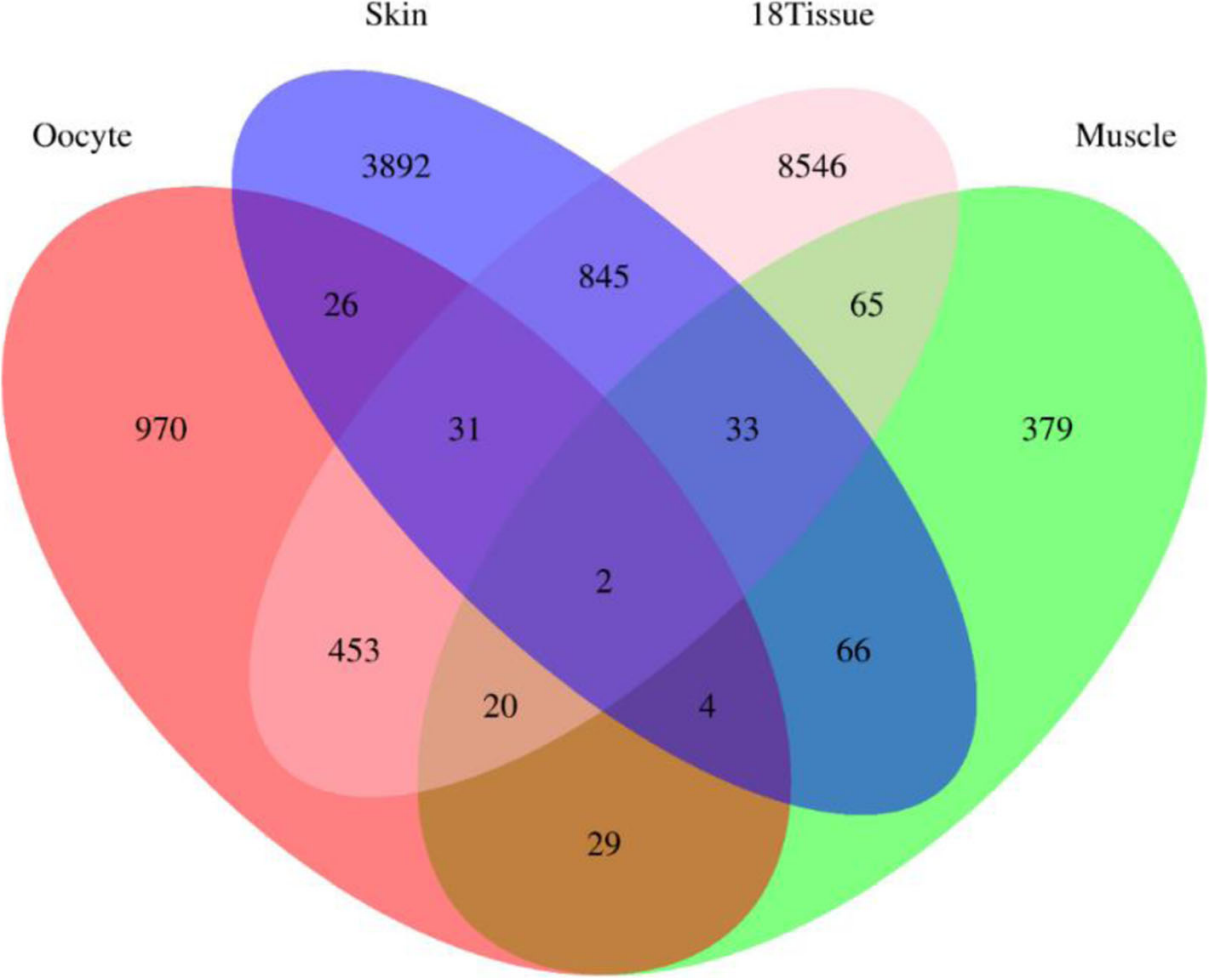
Venn diagram of comparative analysis of oocyte lincRNAs with bovine lncRNAs from similar studies. The green circle represents the lncRNAs found in bovine muscle. The blue circle represents the lncRNAs found in bovine skin. The orange circle represents the lncRNAs identified in 18 bovine tissues that include adrenal gland, black skin, white blood cells, caudal lobe of brain, brain cerebellum, heart, kidney, leg muscle, liver, lung, intestinal lymph node, mammary gland, ovary, spleen, thymus, thyroid, tongue and white skin

Comparison of the genomic position of the 1535 oocyte lincRNAs with the position of previously identified lncRNAs in the NONCODEv4 database showed that 115 (7.6%) of the oocyte lincRNAs identified in this study overlap with previously reported bovine lncRNAs (Additional file 2: Table S2). A comparison of the oocyte lincRNAs with the lncRNAs found in similar studies in cattle was also performed (Fig. 2). Of the 4899 skin lncRNAs [15], 63 were found to overlap with oocyte lincRNAs. Moreover, 55 of the 584 muscle lncRNAs [16] were found to overlap with our putative oocyte lincRNAs. Further analysis revealed that the 9778 lncRNAs identified from 18 bovine tissues [17] include 506 lncRNAs that are present in this collection of oocyte lincRNAs. Notably, only 2 lncRNAs were shared by all four studies. A total of 970 lincRNAs were unique to bovine oocytes, indicating that lncRNAs are expressed in a tissue-specific manner.

### Tissue specificity of bovine oocyte lincRNAs

**Fig. 3:**
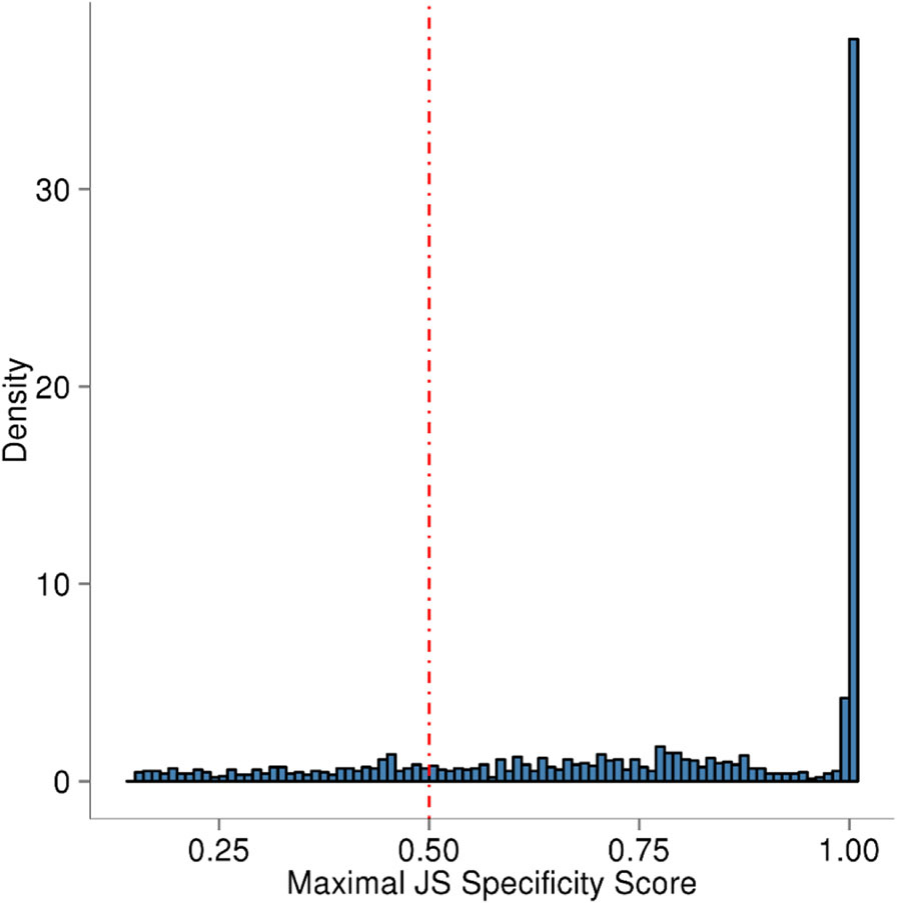
Distribution of maximal JS (Jensen-Shannon) specificity scores of bovine oocyte lincRNAs. The JS scores range from 0 to 1, with 1 being perfect specificity. More than 37% of the oocyte lincRNAs have a JS score of 1, suggesting they are expressed exclusively in bovine oocyte

In order to calculate the tissue specificity score for each oocyte lincRNA, we downloaded RNA-Seq data sets of 9 bovine tissues from NCBI SRA database (Accession number SRR594491- SRR594499). The FPKM (fragments per kilobase of transcript per million mapped reads) value of each transcript in each of the 9 tissues was calculated by Cufflinks (Additional file 3: Table S3). A tissue specificity score for each lincRNA transcript was then calculated using an entropy-based metric that relies on the Jensen-Shannon (JS) divergence [28]. The distribution of JS scores is shown in Fig. 3. Using a JS score of 0.5 as a cutoff [38], the majority of oocyte lincRNAs (80%) are tissue-specific. Notably, more than 37% of the oocyte lincRNAs have a JS score of 1, suggesting they are expressed exclusively in bovine oocyte.

**Fig. 4:**
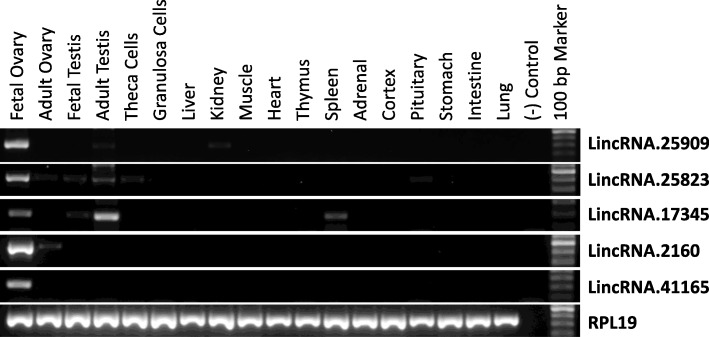
Analysis of tissue distribution of selected oocyte lincRNAs. Expression of 5 most abundantly expressed oocyte lincRNAs (all have a JS score of 1) in bovine tissues was analyzed by RT-PCR. Bovine RPL19 was used as a control for RNA quality

To analyze the tissue distribution of the oocyte lincRNAs, we performed RT-PCR analysis on 5 most abundantly expressed lincRNAs based on the FPKM values (> 150). As shown in Fig. 4, 4 of the 5 selected lincRNAs (except for lincRNA.17345) showed either specific or predominant expression in the fetal ovary (a rich source of oocytes). In particular, expression of lincRNA.2160 and lincRNA.41165 was only detected in fetal/adult ovary but not in a panel of 14 other tissues and ovarian follicular cells (granulosa and theca cells) indicating that they are exclusively expressed in oocytes.

### Functional prediction of bovine oocyte lincRNAs

**Fig. 5:**
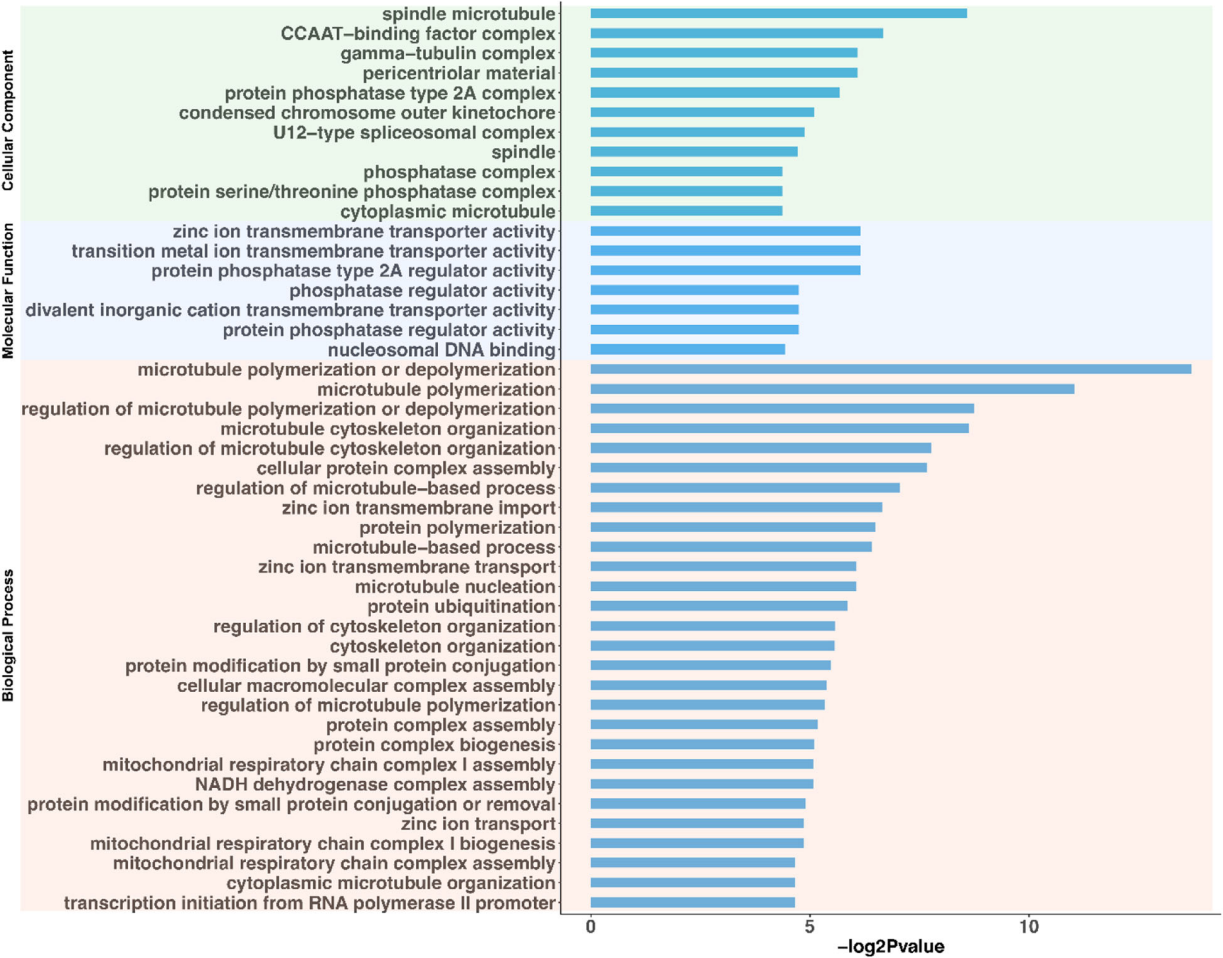
Gene ontology (GO) analysis of the neighboring protein-coding genes of 75 oocyte-specific lincRNAs

Recent studies suggest that some lincRNAs may act in *cis* and regulate the expression of a neighboring protein coding gene [39, 40]. The expectation of the *cis* regulation hypothesis is that the expression between lincRNA and its neighboring gene would be correlated across all samples used in the present study. To predict the potential functions of the oocyte lincRNAs, a total of 1239 lincRNAs with a JS score larger than 0.5 were selected. We then screened ~ 50 kb genomic region as neighboring chromosome region [41] flanking the genomic loci of the 1239 lincRNAs on either direction using BEDTools [42]. A total of 202 mRNAs were identified as “neighbors” of the bovine oocyte lincRNAs. Furthermore, we calculated the Pearson’s correlation coefficients (PCC) between lincRNAs and their neighboring genes and analyzed enriched GO terms associated with mRNAs that are strongly correlated with neighboring lincRNAs. Finally, we identified 75 oocyte-specific lincRNAs strongly co-expressed with 58 neighboring protein-coding genes. As shown in Fig. 5, mitochondrial respiratory chain complex assembly, cytoskeleton organization, protein modification and microtubule-based process were enriched in biological process. Zinc ion transmembrane transporter activity, phosphatase regulator activity and nucleosomal DNA binding were over-represented in molecular function. The enrichment of neighboring genes in cellular component is mostly related to phosphatase complex, condensed chromosome outer kinetochore, CCAAT-binding factor complex, pericentriolar material and spindle microtubule. These results suggest that a portion of bovine oocyte lincRNAs might act locally to regulate their neighboring genes in *cis*.

## Discussion

In this study, we presented the comprehensive annotation of bovine oocyte lincRNAs using RNA-Seq data from bovine oocytes. In order to assess tissue specificity of newly identified lincRNAs in bovine oocytes, we collected RNA-Seq data sets from multiple bovine tissues from NCBI RSA database. The tissue-specificity score was calculated based on the FPKM for each transcript and demonstrated that bovine oocyte lincRNAs are expressed in a much more tissue-specific manner. It would be interesting to find out if oocyte lncRNAs in other species, such as humans, show similar expression pattern. Similar studies could be conducted in humans using RNA sequencing data from human oocytes. Such data have become available recently [43].

Based on the hypothesis that lincRNAs might act in *cis* to regulate the gene expression in their chromosomal neighborhood, we were able to predict the putative functions for 75 oocyte lincRNAs. Most importantly, we found that cytoskeleton organization, regulation of microtubule-based process, zinc ion transport and mitochondrial respiratory chain complex assembly were over-represented for neighboring genes of oocyte-specific lincRNAs. Early embryonic development in many organisms relies on the subcellular organization of the oocyte and requires the coordination of a variety of cellular events. Cytoskeleton was believed to mediate many of these processes. More importantly, microtubules, a component of the cytoskeleton, are the major constituents of spindles that are used to pull apart eukaryotic chromosomes during mitosis and meiosis. Moreover, Stephenson et al. [44] reported that the zinc level would affect bovine oocyte maturation and fertilization in vitro. Furthermore, Kong et al. demonstrated that rapid cellular zinc influx regulates early mammalian development during the oocyte-to-egg transition through modulation of the meiotic cell cycle [45]. Instead of transcriptionally based mechanism, they found that it is two maternally derived zinc transporters that control zinc uptake. Targeted knockdown of these transporters during meiotic maturation perturbs the intracellular zinc quota and results in a cell cycle arrest at a telophase I-like state in mouse oocyte. The importance of mitochondria was highlighted by their crucial role to support critical events such as spindle formation, chromatid separation and cell division during oocyte maturation. It is known that the developing zygote is dependent on the existing pool of mitochondria until blastocyst implantation [46].

Reversible phosphorylation is important in regulating oocyte meiosis. The inhibition of phosphatase-1 (PP1) and PP2A was found to stimulate oocyte germinal vesicle breakdown [47]. Phosphorylation of PP1 at Thr320 by cyclin dependent kinase-1 (CDK1) causes PP1 inactivation. GV-intact oocytes do not contain phosphorylation of Thr320 of PP1. Moreover, inhibition of oocyte germinal vesicle breakdown by roscovitine (ROSC) was shown to coincide with PP1 phosphorylation at Thr320 [48]. Besides, the pericentriolar material (PCM) is a matrix of proteins serving as a platform for spindle assembly [49]. The over representation of PCM, together with the enrichment of condensed chromosome outer kinetochore and spindle microtubule, suggests activity of spindle apparatus assembly. Taken together, these results indicate the involvement of bovine oocyte lincRNAs in oogenesis through regulating their neighboring protein-coding genes.

Oocyte-specific genes are known to play important roles in folliculogenesis, fertilization, and early embryonic development [50]. This study provides a foundation for future investigations on the roles of oocyte-specific lncRNAs in controlling oocyte development and early embryogenesis. Our future studies will be focused on characterizing the expression of these oocyte-specific lncRNAs during oocyte maturation and early embryogenesis, determining their associations with oocyte quality, and evaluating their roles in the regulation of oocyte-expressed genes required for oocyte maturation and development of early embryos in cattle. Such studies would help identify important lncRNAs in human oocytes that could be used as biomarkers for oocyte quality and embryos with high developmental potential as cow is a good model to study oocyte development and early embryonic development for humans [51, 52].

## Conclusions

In the present study, we performed ab initio assembly of more than 80 million RNA-Seq reads from bovine GV and MII stage oocytes and identified 1535 transcribed lincRNAs from 1183 loci. In addition, we calculated the tissue specificity score for each oocyte lincRNA and demonstrated that the majority of oocyte lincRNAs (80%) are tissue-specific. Finally, we proposed functions of oocyte specific lincRNAs, suggesting their involvement in oogenesis through regulating their neighboring protein-coding genes. This study provides a foundation for future investigations on the roles of oocyte-expressed lncRNAs in controlling oocyte development and early embryogenesis in cattle.

## Supplementary information

**Additional file 1: Table S1.** List of lincRNAs identified in bovine oocytes.

**Additional file 2: Table S2.** Comparison of bovine oocyte lincRNAs with previously identified lncRNAs in NONCODEv4 database.

**Additional file 3: Table S3.** Expression of lincRNAs in bovine oocytes relative to 9 other tissues.

## Data Availability

The datasets used and/or analyzed during the current study are available from the corresponding author on reasonable request.

## Abbreviations

FPKM: Fragments per kilobase of transcript per million mapped reads
GV: Germinal vesicle
lincRNAs: long intergenic non-coding RNAs
lncRNAs: long non-coding RNAs
MII: Metaphase II
RT-PCR: Reverse transcription polymerase chain reaction
